# TC-motifs at the TATA-box expected position in plant genes: a novel class of motifs involved in the transcription regulation

**DOI:** 10.1186/1471-2164-11-166

**Published:** 2010-03-12

**Authors:** Virginie Bernard, Véronique Brunaud, Alain Lecharny

**Affiliations:** 1 Unité de Recherche en Génomique Végétale (URGV), UMR INRA 1165 -CNRS 8114 -UEVE, 2 Rue Gaston Crémieux, 91057 Evry Cedex, France; 2 Université Paris-Sud, Institut de Biotechnologie des Plantes (IBP), UMR CNRS 8618 -UPS, Bâtiment 630, 91405 Orsay Cedex, France

## Abstract

**Background:**

The TATA-box and TATA-variants are regulatory elements involved in the formation of a transcription initiation complex. Both have been conserved throughout evolution in a restricted region close to the Transcription Start Site (TSS). However, less than half of the genes in model organisms studied so far have been found to contain either one of these elements. Indeed different core-promoter elements are involved in the recruitment of the TATA-box-binding protein. Here we assessed the possibility of identifying novel functional motifs in plant genes, sharing the TATA-box topological constraints.

**Results:**

We developed an *ab-initio *approach considering the preferential location of motifs relative to the TSS. We identified motifs observed at the TATA-box expected location and conserved in both *Arabidopsis thaliana *and *Oryza sativa *promoters. We identified TC-elements within non-TA-rich promoters 30 bases upstream of the TSS. As with the TATA-box and TATA-variant sequences, it was possible to construct a unique distance graph with the TC-element sequences. The structural and functional features of TC-element-containing genes were distinct from those of TATA-box- or TATA-variant-containing genes. *Arabidopsis thaliana *transcriptome analysis revealed that TATA-box-containing genes were generally those showing relatively high levels of expression and that TC-element-containing genes were generally those expressed in specific conditions.

**Conclusions:**

Our observations suggest that the TC-elements might constitute a class of novel regulatory elements participating towards the complex modulation of gene expression in plants.

## Background

Over the past genomics has markedly changed our view on core-promoter organization [1]. For instance, the TATA-box can no longer be regarded as a general feature of polymerase II core-promoters [2]. Indeed, only a small fraction of eukaryotic genes actually harbour a TATA-box: less than 20% of genes in both human [3] and yeast [4]. While TATA-box-containing promoters support the direct binding of TATA-box-Binding Proteins (TBPs) and thereby also the formation of the pre-initiation complex, TATA-less promoters are recognized by multiple TBP-related proteins and other TBP-associated Transcription Factors (TFs) involved in the recruitment of TBP [5]. Indeed some TATA-variants and other alternative elements allow the initiation of transcription and participate towards defining distinct patterns of expression [4,6-9].

Several core-promoter elements or general Transcription Factor Binding Sites (TFBSs) have been previously identified in eukaryotes. They are characterized by a strong positional preference relative to the Transcriptional Start Site (TSS) as for instance the TATA-box in the [-30, -25] area [8], the Initiator element, (Inr), around the TSS [10], the downstream promoter element in the [+28, +33] area [11], or the IIB recognition element immediately upstream of certain TATA-boxes [12]. The position of binding sites of proteins belonging to the transcription complex is important for the functioning of promoters since it determines both the TSS location [13] and the transcription direction [5]. Thus, a strong positional conservation of a novel regulatory element would strongly indicate a functional role. This concept has led to a generation of tools that in contrast to previous TFBS predictors [14,15] are based on the positional densities of oligonucleotides rather than on their frequency of occurrence. These tools have been used to characterize core-promoter elements in several model genomes including plants [16-22].

All together, the core-promoter elements listed above seem unable to account for the transcription of all the RNA-polymerase-II transcribed genes. Less conserved core-promoter elements present in small gene sets have been described in previous studies at the gene level. For instance, in the human cytosolic phospholipase A2-alpha gene, an AAGGAG motif in the [-35, -30] area binds TBP and is critical for basal transcriptional activity [23]. In other studies, a TBP has been shown to bind to a TAAGAGA element in the [-23, -17] region of the hepatitis B virus S gene [24]. These experimental observations suggest that core-promoter elements specific to small sets of genes remain to be disclosed. A study of large-scale structural properties of DNA in promoters indicated that the instability of DNA around -30 relative to the TSS necessary for transcription may be due to as yet unidentified motifs other than the TATA-box [25].

It is therefore clear from and despite this growing amount of data that the code embedded within core-promoter sequences has not yet been fully deciphered. In this work, we used an *in silico *hypothesis-driven approach to predict novel elements potentially recognized by the transcriptional complex. We explored the bioinformatics-based evidence that sequences other than the TATA-box and TATA-variants but located in the same region relative to the TSS may be functional core-promoter elements. We therefore searched for short sequences exhibiting similar positional constraints to those of the TATA-box and identified pyrimidine-rich elements distinct from the pyrimidine tract [21,26] as candidate elements for about 18% of the plant genes. To determine their potential functional role, we investigated any association between such identified TC-elements and specific features of the genes containing them, as has been previously shown for the TATA-box.

## Results

### Less than 39% of *A. thaliana *promoters contain a TATA-box or a TATA-variant

Our approach was based on three steps. First, we searched for all 6 base long motifs with a statistically significant preferential position within the 300 nucleotides upstream of the TSS and called these motifs the Preferentially Located Motifs (PLMs). This method was first described by FitzGerald *et al*. [27] for the analysis of human promoters and was then applied by others to plant promoters [19,21]. Our results correlated well with previous studies in terms of the spatial representation of the PLMs within the plant promoters. Second, for each PLM, we precisely defined: (i) the preferential position relative to the TSS, *i.e*. the top of the peak, (ii) the functional window, *i.e*. the peak width, derived from the peak boundaries, (iii) the Score of Maximal Square relative to the base line (SMS, see Methods section) ranking the PLMs in terms of their topological constraint and (iv) the list of genes containing the studied PLM within the functional window. Third, to increase the chance of identifying functional PLMs, we searched for their conservation in 14927 *A. thaliana *genes and 18012 *O. sativa *genes with experimentally supported TSSs. A well-annotated genomic sequence is available for both of these species that diverged approximately 150 million years ago [28,29]. As previously described within the 50 nucleotides upstream of the TSS of both *A. thaliana *[19,21] and *O. sativa *[26] we found PLMs made up exclusively or almost exclusively of: (i) T and A nucleotides and exhibiting strong topological constraints, *i.e*. a sharp peak, and (ii) T and C nucleotides and exhibiting low topological constraints, *i.e*. a wide peak. Among the T and A rich motifs, we found the canonical TATA-box defined by the TATAWA consensus (W for A or T) and TATA-box variants [8]. We wondered whether by analysing the whole promoter set, other motifs with the same strong topological constraints could be missed. It is reasonable to predict that the presence of a PLM characterized by a wide functional window overlapping the TATA-box expected area might hide the strong topological constraints of a PLM specific to a small promoter set. To address this issue, we built up promoter sets by successively subtracting from the whole set of promoters, those characterized by different classes of PLMs, as described below. We firstly considered the conservation of PLMs at the genome level and then their conservation at the orthologues level.

**Figure 1:**
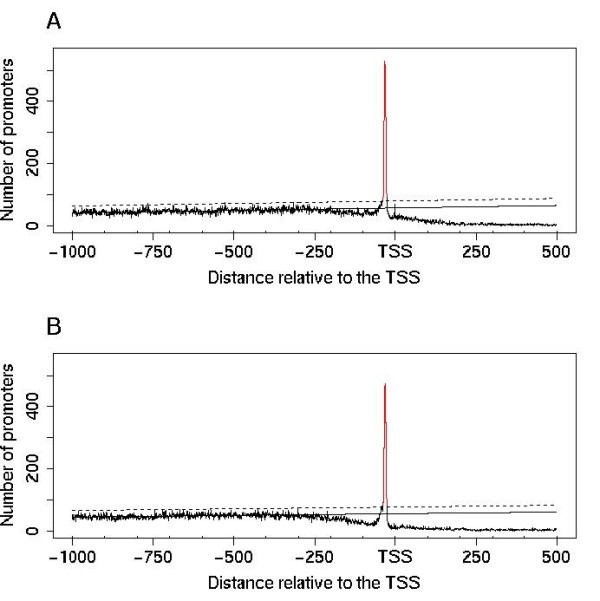
**Distribution of TATAWA in plant promoters aligned relative to the TSS**. The canonical TATA-box defines the TATAWA-PLM. The distribution model is learned in the [-1000, -300] region where the base line (continuous line) and the upper bound of the confidence interval (dashed line) are estimated and applied to the [-300, 500] region. Distributions are shown for the *Arabidopsis thaliana *set containing 14927 promoters (A) and for the *Oryza sativa *set containing 18012 promoters (B). The TATAWA-PLM is preferentially positioned 32 bases upstream of the TSS with a [-39, -26] functional window in both genomes. *A. thaliana *and *O. sativa *are characterized by an SMS of 21 and 19 respectively.

TATAWA is a particularly well-conserved PLM since it is found in the same promoter region in both plants and animals [30]. Confirming previous results, we found this PLM in both *A. thaliana *and *O. sativa *genomes in a preferential position 32 bases upstream of the TSS and strictly located within the [-39, -26] region (Figure 1). A total of 2606 (17.5%) and 2601 (14.4%) promoters in *A. thaliana *and *O. sativa *respectively contained a TATAWA within the [-39, -26] functional window. In contrast to the motif counting method highly prone to false positives, our method may slightly underestimate the number of functional motifs. Indeed, as our aim was to characterize and compare different sets of genes each containing defined PLMs (TATA-box or other) within their promoters, we chose only "clean" sets, *i.e*. those containing only one regulatory element to the detriment of completeness.

**Figure 2:**
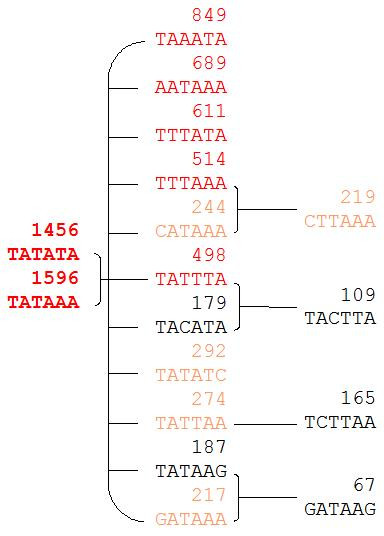
**Sequence distance graph of TA-PLMs present in *A. thaliana *promoters**. The TATA-box PLM sequence (first column), TATAΔ1-PLM sequences (second column) and TATAΔ2-PLM sequences (third column) are organized in an oriented graph. In red the motifs observed in more than 350 promoters; in orange the motifs observed in more than 200 promoters and in black the motifs observed in up to 200 sequences. Each edge between two PLMs reflects the presence of one substitution leading from one PLM to another one. Numbers of *A. thaliana *promoters containing a given motif are indicated above the sequences. The TATAWA motifs are seeds (in bold).

Several T and A rich motifs partially matching the TATAWA sequence have been shown at the TATA-box expected position [31,32]. We observed PLMs with the same positional constraints as TATAWA, *i.e*. showing a sharp peak in the [-39, -26] region. These PLMs may be (i) functional motifs hereafter named as TATAΔ-PLMs where Δ stands for variant; or (ii) motifs being PLMs due to their overlap with the TATAWA sequence. This overlap represents an inherent drawback of the method since motifs shorter than 6 nucleotides (TAT for instance) as well as 6 base long motifs (NNTATA for instance) may appear as PLMs. TATA-variant and TATA-box are distinct regulatory elements that need differentiating [33]. For this reason, we searched for TATAΔ-PLMs in sets of promoters not containing the canonical TATA-box in the [-39, -26] region. In this way, we were able to ascertain that the PLMs found within the promoter sets were *bona fide *elements under topological constraint. In *A. thaliana *and *O. sativa *respectively 12321 (82.5%) and 15411 (85.6%) promoters were found without a TATAWA in the [-39, -26] region. Relative to the TATAWA sequence, 32 possible motifs diverged at one position. Out of these 32 motifs, only 11 were found to represent PLMs (TATAΔ1-PLMs) conserved in both species (Figure 2, column 2). Consistent with *in vivo *experimental results [34], T to A or A to T substitutions were the main differences observed among the TATAΔ1-PLMs. Motifs with a base C or G substitution at the central positions were not considered as PLMs and were counter-selected. Motifs with a C or G substitution at the T1 or A6 positions were, however, considered as TATAΔ1-PLMs. There remained 9512 (63.7%) and 13494 (74.9%) of *A. thaliana *and *O. sativa *genes respectively containing neither a TATAWA nor a TATAΔ1-PLM. We identified only 4 conserved TATAΔ2-PLMs amongst the 211 possible motifs (Figure 2, column 3) and no conserved TATAΔ3-PLM. Finally, at the TATA-box expected position, 4151 *A. thaliana *(27.8%) and 3045 *O. sativa *promoters (16.9%) contained a TATAΔ-PLM. Hereafter, TATAΔ- and TATAWA-PLMs are collectively referred to as TA-PLMs.

**Figure 3:**
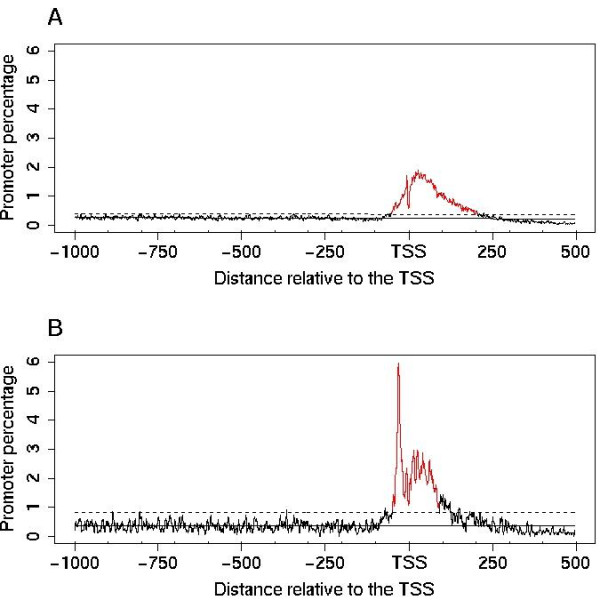
**Distributions of TTCTTC in *A. thaliana *promoters aligned relative to the TSS**. TTCTTC is one of the 29 conserved TC_[-39,-26]_-PLMs. Distributions are shown for the whole promoter set containing 14927 promoters (A) and for the 1745 TA-less promoters (B). The y-axis percentage depends on the promoter number in each data set. TTCTTC is preferentially positioned at +29 (SMS 10) and -33 (SMS 8) in the whole promoter set and in the TA-less promoter set respectively. In both cases, the size of the window used to scan the promoters was 2 bases.

The number of genes containing the TA-PLMs decreased from the TATA-box to the TATAΔ2-PLMs (Figure 2). Interestingly, even though most of the TATAΔ-PLMs were found to be T and A rich as expected, 3 out of the 4 TATAΔ2-PLMs (CTTAAA, TACTTA and TCTTAA) contained a base C. This suggested that some T and C rich PLMs, hereafter referred to as TC-PLMs, could have been missed in the analysis of the whole promoter set. Indeed, it has been shown that, in plants, the region overlapping the TSS is characteristically rich in T and C nucleotides described as CpT microsatellites or TC-microsatellites [35,36]. We observed these microsatellites during the analyses of the whole promoter set within a number of different 6-base-long PLMs. They are characterized by a wide functional window centred downstream of the TSS often extending several hundred bases. We sometimes observed a small secondary peak located around 30 bases upstream of the TSS which disturbed the broad distribution of some of the TC-microsatellites (Figure 3.A). These TC-microsatellites may hide TC_[-39,-26]_-PLMs, *i.e*. TC-PLMs with high topological constraints similar to those of TA-PLMs present within specific sub-sets of genes.

### Conserved TC_[-39,-26]_-elements are observed in almost 18% of *A. thaliana *promoters

We hypothesised that TC_[-39,-26]_-PLMs with narrow functional windows could be functional alternatives to TA-PLMs. This being the case we expected to be able to detect these PLMs more easily in promoter sets within which they predominate or within promoters not containing TA-PLMs. We therefore selected a TA-less promoter set by excluding the TA-PLM-containing promoters from the whole promoter dataset. Out of the 16 possible dinucleotides, only 3 were found to be conserved PLMs and to shared the TATA-box topological constraints: TA, AT and AA. A total of 1745 *A. thaliana *promoters (11.7%) and 5089 *O. sativa *promoters (28.2%) contained no TATA-box or conserved TATAΔ-PLM or the 3 conserved dinucleotide-PLMs.

We examined the possibility of these TA-less promoter sets representing sets of poorly annotated promoters due to an incorrect prediction of the TSS position. In both TA-less promoter sets, three arguments supported the validity of the TSS position prediction. First, the observed TC_[-39,-26]_-PLMs were conserved between *A. thaliana *and *O. sativa *and in both plants they exhibited the same strict topological constraints (see below). Second, the distributions of several motifs known as TFBSs supported by experimental analyses [37,38], such as the Inr or the GGCCC element, shared the same topological constraints in both the whole and the TA-less promoter sets (data not shown). Third, as expected, the GC-compositional strand bias or GC-skew expected in plant promoters was observed at the TSS location in both promoter sets (data not shown).

We therefore searched in the TA-less promoter sets for the presence of 6-base-long conserved PLMs. Out of the 4096 possible motifs, we found 29 conserved PLMs exhibiting a strict functional window in the [-39, -26] area relative to the TSS. These PLMs were present in 2645 *A. thaliana *(17.7%) and in 2331 *O. sativa *(12.9%) promoters. In agreement with our hypothesis, the 29 PLMs were TC_[-39,-26]_-PLMs, *i.e*. were comprised of T and C bases only. Figure 3 illustrates how the TC_[-39,-26]_-PLMs can be clearly distinguished in the TA-less promoter set while they were missed in the whole promoter set. Indeed, using the whole promoter set, it is only possible to find the TTCTTC-PLM, which is characterized by a wide functional window and by a preferential position 29 bases downstream of the TSS (Figure 3.A). On the contrary, using the TA-less promoter set, we observed both the wide functional window PLM and a distinct TTCTTC-PLM characterized by a sharp peak centred 33 bases upstream of the TSS, *i.e*. sharing the TA-motif topological constraints (Figure 3.B). We propose that TA-PLMs may be recognized and thus be functional in a T and C rich environment whereas the same might not be true for TC_[-39,-26]_-PLM. As a consequence, large pyrimidine-rich regions could have been preferentially maintained during evolution in promoters with a TA-PLM whereas they may have been counter-selected, at least upstream of the TSS, in promoters with a functional TC_[-39,-26]_-PLM. It is important to note that only TC_[-39,-26]_-PLMs exhibited the TATA-box topological constraints in the TA-less promoter set. Altogether, our results (i) confirmed the importance of TC-microsatellites (TC_{*n*} _or TTC_{*n*} _for instance - Figure 3.A) in plant promoters and (ii) predicted the existence of a novel class of functional elements, the TC_[-39,-26]_-PLMs characterized by a sharp peak in the [-39, -26] region and observed in almost 18% of *A. thaliana *promoters.

We investigated the putative presence of TC_[-39,-26]_-PLMs in other eukaryotic genomes. We analyzed 15802 *Homo sapiens *promoters and 15833 *Mus musculus *promoters with an experimentally supported TSS [39]. We observed neither TC_[-39,-26]_-PLMs nor any other PLMs at the TATA-box expected region. Thus, both the TC-microsatellites and the TC_[-39,-26]_-PLMs observed in plants are absent in both human and mouse. These observations suggest an evolutionary link between the presence of the TC_[-39,-26]_-PLMs and the TC-microsatellites.

### TC_[-39,-26]_-elements derived from three seed motifs

As for all identified TATAΔ-PLMs, any 6-base-long TC_[-39,-26]_-PLM may be a functional PLM, be a part of a larger functional PLM or contain a smaller functional PLM. Any of the TC_[-39,-26]_-PLMs can potentially overlap by 5 consecutive bases with at least one other TC_[-39,-26]_-PLM. Among the promoters studied, we found only 3 overlapping TC_[-39,-26]_-PLMs: CTTCTT, TTCTTC and TCTTCT. The trinucleotide repetition made up of 2 T bases and one C base is characteristic of self-overlapping motifs involved in DNA recognition by transcription factors [40]. Furthermore, we analyzed the effect of extending each of the 29 TC_[-39,-26]_-PLMs on SMS, *i.e*. the distribution score. Extended PLMs with higher SMS scores than those of the initial 6-base-long PLM were considered to be functional candidates. CTTCTT and TTCTTC could be extended up to the 9-base-long TCTTCTTCT PLM that exhibited the highest SMS (Additional file 1). Together, these results provide evidence in favour of the TCTTCTTCT motif being a functional TC_[-39,-26]_-PLM. Extension of most of the other TC_[-39,-26]_-PLMs did not generate PLMs with higher SMS than the SMS of the initial 6-base-long PLM. Note that the TC-microsatellites observed in the gene 5' UnTranslated Regions (UTRs) could be extended up to a 13-base-long PLM (data not shown). The shortest core-PLMs were CTC, TCT and CTT, found within 27 TC_[-39,-26]_-PLMs, suggesting that these trinucleotides could be the core of the functional TC_[-39,-26]_-PLMs. Both the trinucleotide repetitions and the different T and C environments might therefore have a role in TC_[-39,-26]_-PLM function.

**Figure 4:**
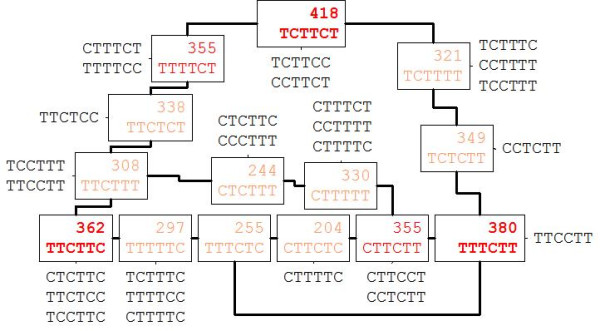
**Sequence distance graph of the TC_[-39,-26]_-PLMs present in *A. thaliana *promoters**. For each TC_[-39,-26]_-PLM observed in more than 200 *A. thaliana *promoters, the number of promoters containing the PLM is indicated above the sequence (surrounded data). In red PLMs observed in more than 350 promoters; in orange, PLMs in more than 200 promoters and in black PLMs observed in up to 200 sequences. TC_[-39,-26]_-PLMs observed in more than 200 promoters are organized in an oriented and closed graph. Each edge between two PLMs reflects the presence of one substitution leading from one PLM to another. Edges between the less observed PLMs (in black) are not considered. The three seeds of this graph (in bold) are PLMs of which all the directly connected motifs are less frequently observed than the seed-PLMs themselves. Only one graph is possible.

Three observations are in favour of a putative role of most TC_[-39,-26]_-PLMs independently of the presence of other PLMs in the same region. First, TC_[-39,-26]_-PLMs were often observed without any of the other PLMs sharing the same preferential position in [-39, -26]. Second, TC_[-39,-26]_-PLMs are not extensions of either a TATA-box or its variants. Thus, among the _[-39,-26]_-PLM-containing promoters, 80% contained PLM-motifs belonging to only one PLM class, either a TATA-box, a TATAΔ- or a TC_[-39,-26]_-PLM. Third, it was possible to construct oriented graphs displaying the motif divergence for both TA- and TC-PLMs (Figure 2 and 4). Concerning the TA-PLMs, the graph root or seed is the TATAWA-PLM. For a given TA-PLM, the number of promoters containing it depends on both the distance from the seed, *i.e*. the number of substitutions between the given PLM and the seed-PLM and the existence or not of a more divergent sequence. We applied the same approach to the conserved TC_[-39,-26]_-PLMs and found that they could be organized into a unique, closed and oriented graph (see Methods section). Three seeds were suggested: TCTTCT, TTTCTT and TTCTTC (Figure 4). Similar to that observed for TA-PLMs, these three TC_[-39,-26]_-PLMs were those most frequently observed in promoters with the number of promoters containing the other TC_[-39,-26]_-PLMs depending on both the distance from these seeds and the existence or not of a more divergent sequence. In conclusion, all _[-39,-26]_-PLMs can be detected independently in different promoters and may be organized into different groups with apparent evolutionary links between the given PLM and the related seed.

### TC_[-39,-26]_-element-containing genes are preferentially involved in protein metabolism

**Figure 5:**
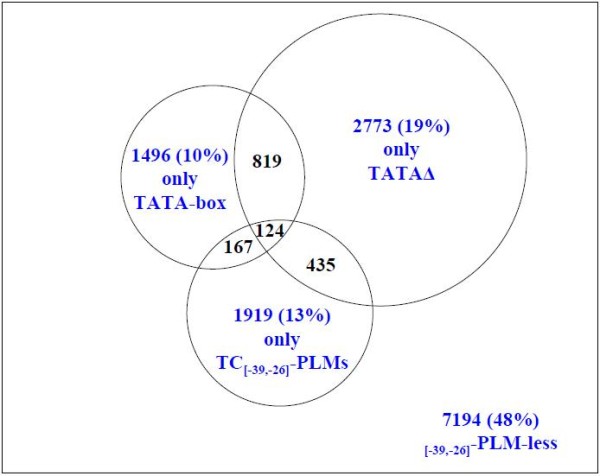
**Presence in *A. thaliana *promoters of the three PLM classes characterized by a sharp functional window and preferentially located in the [-39, -26] region**. The square represents the 14927 *A. thaliana *promoters (the whole promoter set) with experimentally known TSS. The central Venn diagram illustrates the overlaps between the three PLM classes in promoters. In the [-39, -26] region, 2606 promoters contained a canonical TATA-box, 4151 a TATAΔ-PLM and 2645 a TC_[-39,-26]_-PLM. In black the overlap between the three classes. In blue, the promoter sets analysed in the study, *i.e*. the promoter sets with PLMs from only one of the three classes and the promoter set that does not contain PLMs of the three classes.

While most promoters contain only one class of _[-39,-26]_-PLM, some contain more than one (Figure 5). To investigate the functional significance of the different _[-39,-26]_-PLM classes we decided to construct four promoter sets each characterized by the exclusive presence of one of the classes of PLM in the [-39, -26] region. We distinguished (i) the 1496 promoters containing only a TATA-box, (ii) the 1919 containing only a TC_[-39,-26]_-PLM, (iii) the 2773 containing only a TATAΔ-PLM and (iv) as a negative reference the 7194 promoters without any _[-39,-26]_-PLM, called hereafter the _[-39,-26]_-PLM-less set.

Gene Ontology (GO) annotations [41] have been used previously in the prediction of the functional role of TFBS [42]. Here we analysed the GO annotations of the four classes of genes defined above, according to the four promoter sets. As far as biological processes are concerned, the most conspicuous observation was a preferred association between genes containing a TATA-box and responses to different stimuli (Table 1, Biological Process). This result was expected since in human and in yeast the TATA-box-containing genes are more frequently involved in specific biological processes [4,9]. Interestingly, we also found that TC_[-39,-26]_-PLM-containing genes are more frequently involved in protein metabolism than any other gene class (Table 1, Biological Process), *i.e*. a basic biological process. Neither the TATAΔ-containing genes nor the _[-39,-26]_-PLM-less genes showed any significant bias for any biological process categories. We also analysed the partitioning of genes between the different GO cellular components. Again, the TATA-box-containing genes presented the most biased partitioning. The products of these genes are more often constituents of the cell wall, the cytosol and the ribosomes and less frequently constituents of the chloroplasts, the plastids, the Golgi apparatus and the mitochondria compared to the products of all the other genes (Table 1, Cellular Components). Products of the TC_[-39,-26]_-PLM-containing genes exhibited little biased partitioning between the GO cellular component categories. Indeed, only the cell wall component was significantly less represented. It seems biologically sound to have found a positive correlation between the response to different stimuli and the cell wall GO categories in the TATA-box-containing genes and a negative correlation between the protein metabolism and the cell wall categories for TC_[-39,-26]_-PLM-containing genes. These observations suggest that TC_[-39,-26]_-PLMs might have a functional role in transcriptional control that could frequently differ from and perhaps even oppose the TATA-box functional role. It is of particular interest that our observations support the hypothesis that small variations in the TATA-box sequence are linked to large changes in gene expression [6,43]. Unfortunately, due to the relatively small number of promoters containing a unique TC_[-39,-26]_-PLM, it was deemed impossible to perform the same comparisons between the TC_[-39,-26]_-PLM seeds alone and their predicted variants as that performed for the TA-motifs. It should be noted that by analysing all the genes containing a TC_[-39,-26]_-PLM alone we decreased the power of our statistical tests. We were however able to demonstrate a significant difference between the TATA-box and the TC_[-39,-26]_-PLM sets concerning protein metabolism and cell wall categories (p. values of 3e^-5 ^and 1e^-9 ^respectively).

**Table 1:** Functional category profiles of *A. thaliana *four gene sets.

Gene sets		All genes	Only TATA-box	Only TC_[-39,-26]_-PLMs	Only TATAΔ-PLMs	[-39, -26]^-^-PLM-less
GO Biological process	Response to abiotic or biotic stimulus	8.8	**12 (7e^-4^)**	7.7 (NS)	7.7 (NS)	8.1 (NS)
	Response to stress	8.8	**11 (1e^-3^)**	8.6 (NS)	8.2 (NS)	7.7 (NS)
	Protein metabolism	15	12 (NS)	18 (1e^-3^)	16 (NS)	15 (NS)

GO Cellular component	Cell wall	3.1	**5.8 (1e^-6^)**	1.8 (6e^-4^)	3.1 (NS)	2.7 (NS)
	Ribosome	2.6	**4.2 (1e^-3^)**	1.8 (NS)	3.1 (NS)	2.4 (NS)
	Cytosol	2.8	**5 (4e^-5^)**	1.9 (NS)	3.2 (NS)	2.4 (NS)
	Golgi apparatus	1.4	0.3 (3e^-5^)	1.3 (NS)	1.8 (NS)	1.6 (NS)
	Mitochondria	5.6	3.2 (3e^-5^)	4.8 (NS)	6 (NS)	6.6 (NS)
	Plastid	6.1	3.6 (6e^-5^)	5.2 (NS)	7 (NS)	7 (NS)
	Chloroplast	15	11 (5e^-6^)	15 (NS)	16 (NS)	17 (NS)

Gene functional categories have been retrieved from the GO categories [60] provided by TAIR [41]. In the table, each number not in parenthesis is the percentage of genes annotated in a given GO category. We performed the two one-sided Fisher exact tests allowing the identification of enrichment (bold) or impoverishment (underlined) of GO categories in a given set of genes when compared with all the other genes, *i.e*. genes within the whole gene set minus genes within the considered gene set. NS indicates a non-significant difference between percentages. P-values in parenthesis are less than 5% with the Bonferroni correction. Results are only shown for GO categories exhibiting at least one bias.

### Only the canonical TATA-box is spatially linked to the Initiator element

Recent progress in mammalian genomics has defined a more functional image of core-promoters. Two functional categories of core-promoters have been described [44]. The single peak promoters have a tightly defined TSS position within a few base pairs while in broad peak promoters several TSSs may be found within small clusters spanning tens of base pairs. A TATA-box is more likely to be found within single peak promoters than in broad peak promoters [44]. In genome-wide studies, only the most upstream TSSs are often considered and are defined by the available transcript sequences. There are three main categories of core-promoters described in human [9], *i.e*. TATA-box-containing promoters with an Inr, TATA-box-containing promoters without Inr and TATA-less promoters. We observed the same relative representation of these three categories in *Arabidopsis*. Consistent with mammalian studies [44], recent evidence suggests that *A. thaliana *genes containing a TATA-box tend to have a sharper dominant peak of TSS compared to that of other genes [45].

**Figure 6:**
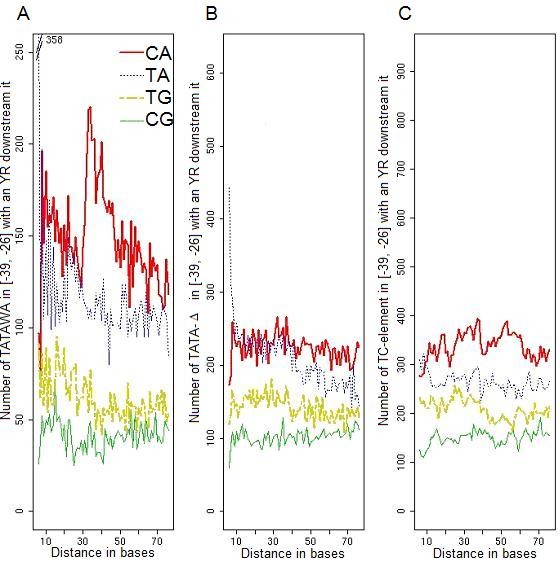
**Distance between the four YR-TSSs relative to the three classes of _[-39,-26]_-PLMs in *A. thaliana *promoters**. We computed the distance observed between each PLM of a given class of _[-39,-26]_-PLMs and all dinucleotides CA (red line), TG (yellow line), TA (blue line) and CG (green line) present downstream of the PLMs. The distance on the x-axis corresponds to the number of bases between the first base of a _[-39,-26]_-PLM and the first base of an YR-TSS. For instance, a distance of 8 bases between a TATAWA and a CA corresponds to the TATAWANNCA sequence in a promoter. We analysed the three gene sets containing a unique class of _[-39,-26]_-PLMs and represented the distance between (A) the TATAWA-, (B) the TATAΔ-, (C) the TC_[-39,-26]_-PLMs and the four YR-TSS sequences. Analyses done for each PLM led to the same representation as the global analysis done for the PLM class it belongs to: for instance, the analysis for the TTCTTC-PLM led to a (C) representation.

The distance between TATA-box and Inr is important for accurate transcription initiation [46]. We considered the distance between the Inr [21], called here YR-TSS, and the conserved PLMs characterized using our approach. As expected, the YR-TSS is a conserved PLM whose functional window is one base upstream of the TSS in both plants (data not shown). We first analysed the 1496 *A. thaliana *promoters containing a TATA-box and we represented the distances between each TATAWA in the [-39, -26] region and each dinucleotide CA, TA, TG and CG up to 70 bases downstream of the TATAWA (Figure 6.A). The dinucleotide CA, but not TA, TG or CG showed a strong preferred distance of 30 to 33 bases from TATAWA. This distance is consistent with the preferential position of both PLMs and with mammalian results [47]. Furthermore, none of the YR motifs showed a preferential distance either from TATAΔ- or from TC_[-39,-26]_-PLMs (Figure 6.B and 6.C). Finally, our results provide evidence of a link between the canonical TATA-box and the CA-TSS: 30 to 33 bases preferentially separate the CA from the first T of TATAWA.

### Genes containing the TC_[-39,-26]_-element are generally long

In human, the presence of a TATA-box in promoters is often associated with a compact gene structure [6]. In *A. thaliana*, we observed the same bias towards a more compact structure of TATA-box-containing genes (Table 2). In contrast, the TC_[-39,-26]_-PLM-containing genes and the _[-39,-26]_-PLM-less genes were overall relatively longer while the gene structure of TATAΔ-PLM-containing genes showed no bias. When present, the bias in gene size was mainly explained by the 5'UTR and by the length of the coding sequence. However, to a lesser extent, the enrichment in compact genes amongst those containing a TATA-box may be explained by the shorter cumulative length of introns, and by the higher percentage of intronless genes (Table 2).

**Table 2:** Structural gene features of *A. thaliana *genes for the four promoter classes.

Gene sets		All genes	Only TATA-box	Only TC_[-39,-26]_-PLMs	Only TATAΔ-PLMs	_[-39,-26]_-PLM-less
Length median	Gene	2293	1936 (5e^-23^)	**2385 (8e^-8^)**	2293 (NS)	**2334 (2e^-7^)**
	5'UTR	158	112 (7e^-38^)	**175 (3e^-5^)**	161 (NS)	**173 (9e^-10^)**
	CDS	1086	966 (5e^-14^)	**1185 (3e^-10^)**	1071 (NS)	**1119 (1e^-4^)**
	All introns	588	521 (1e^-4^)	605 (NS)	588 (NS)	614 (NS)

Percentage	Intron-less	18.8	**24.5 (4e^-9^)**	16.7 (NS)	17.5 (NS)	17.1 (NS)

Structural gene features have been assigned by querying the FLAGdb^++ ^database [59]. For median length data, we performed two one-sided Wilcoxon tests allowing the identification of enrichment in wide (bold) or in compact gene structures (underlined) in a set of genes compared with all the other genes, *i.e*. genes within the whole gene set minus genes within the considered gene set. For intron-less gene percentages, we performed two one-sided Fisher exact tests allowing the identification of higher (bold) or lower (underlined) percentages in a gene set in comparison with all the other genes. NS indicates a non-significant difference. P-values in parenthesis are less than 5% with the Bonferroni correction. Both the first intron and 3'UTR lengths are never biased (data not shown).

Based on the counting of Expressed Sequence Tags (ESTs) [4] or on microarray data [6], gene size and gene expression levels have been shown previously to be inversely correlated in both human and yeast. This being the case, our observations could be due to the fact that, in general, TATA-box genes have higher expression levels than other *A. thaliana *genes [19,21]. The differences in gene sizes we observed are consistent with a lower mean expression of TATAΔ-PLM- than of TATA-box-containing genes. Both TC_[-39,-26]_-PLM-containing and _[-39,-26]_-PLM-less genes could be expressed at lower levels. TATA-box-containing genes have been shown to generally display relatively high specificity of expression compared to housekeeping genes that frequently show high expression levels (Table 1). Therefore, the question remains as to understand whether the presence of regulatory elements in the [-39, -26] region of promoters is linked directly to gene function *per se *or to the transcription level that in turn is associated with gene function. To resolve this we analysed a large set of transcriptome data, distinguishing the two different components of transcription: specificity and level.

### Specific role played by each class of regulatory element found in the [-39, -26] region on different components of transcription

**Figure 7:**
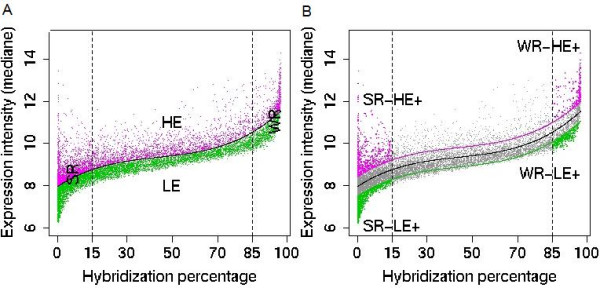
**Subsets of *A. thaliana *genes characterized by different expression intensities and specificities**. Each gene is plotted according to the percentage of plant samples by which the gene specific probe has been hybridized (x-label) and to the median of expression intensities observed in the different samples (y-label). (A) First, we estimated the base line for all data (in black) and we characterized two gene groups: the highly expressed genes (HE, in magenta, 4371 genes) above the base line and the lowly expressed genes (LE, in green, 6790 genes) below the base line. Second, we identified two inflections in the base line corresponding to 15% and 85% of hybridizations (vertical dashed black lines). These limits characterized two extreme sets of genes, the genes exhibiting the Smallest Range of hybridizations (SR, 4510 genes) and the gene exhibiting the Widest Range of hybridizations (WR, 1241 genes). (B) From the SR and WR gene sets, we defined 4 sets of genes with extreme hybridization intensities by estimating an upper (magenta line) and lower (green line) bound at the 60% confidence interval. Thus, there are 618 SR-HE+, 822 SR-LE+, 244 WR-HE+ and 385 WR-LE+ genes.

In eukaryotic genomes, various TFBSs have been linked to specific gene expression patterns [4,6,9,48]. We searched for such a link with PLMs in the [-39, -26] region using *A. thaliana *transcriptome data obtained with the Complete *Arabidopsis *Transcriptome MicroArray (CATMA) [49] and available through the Complete *Arabidopsis *Transcriptome database: CATdb [50]. The dataset included 1044 hybridizations based on 522 different samples covering numerous developmental stages, biotic and abiotic stresses and mutants. We used normalized data on which positive hybridizations have previously been determined (see Methods section in Aubourg *et al*., [51]). For any one gene, the relative number of positive hybridizations was considered as a measure of the range of expression, *i.e*. of the specificity. To measure the global level of expression we computed the median of the signal intensities. The relationship between the median signal intensity and the percentage of hybridizations was clearly not linear and suggested the existence of different classes of genes with respect to transcriptional regulation. We thus clustered the transcriptome data into four classes (Figure 7 and Table 3, first rows). First, genes were separated in two classes depending on their expression level above or below the distribution model line, respectively HE for those with High levels of Expression and LE for those with Low levels of Expression. Second, two classes were defined relating to the specificity of hybridizations and delimited by the two inflections in the cloud of points. The first class clustered genes positively hybridized in less than 15% of microarray hybridizations (SR for Small Range) and the second class clustered those with more than 85% of hybridizations (WR for Wide Range). Interestingly, each of these four transcriptional clusters was predominantly made up of genes containing one of a specific class of _[-39,-26]_-PLMs. Thus, (i) genes within the transcriptional SR group, *i.e*. those that hybridized in specific conditions predominantly contained TC_[-39,-26]_-PLM; (ii) genes within the transcriptional WR group, *i.e*. housekeeping genes predominantly contained a TATAΔ-PLM; (iii) the transcriptional HE group was enriched with the TATA-box-containing genes and (iv) the transcriptional LE group was enriched with the _[-39,-26]_-PLM-less genes. These results clearly indicate a link between the different motifs within the core-promoter and transcriptional features of the gene and are in favour of a functionally independent role of the TATA-box, the TATAΔ- and the TC_[-39,-26]_-PLMs. Our observations provide further evidence for the involvement of the TATA-box in the regulation of transcription level. In addition, our results suggest that both TATAΔ- and TC_[-39,-26]_-PLMs might be involved in defining the specificity of transcription. The role of the TATAΔ-PLMs might be to promote a relatively wide range of expression while on the other hand TC_[-39,-26]_-PLMs may impose narrower limits. The adverse effect of TATAWA and _[-39,-26]_-PLM-less within genes on the extreme level of transcription (see Methods section) was particularly evident in the SR group (Table 3, last two rows). The most Highly Expressed genes (HE+ genes) were predominantly those containing TATAWA whereas the most Lowly Expressed genes (LE+ genes) were mainly _[-39,-26]_-PLM-less genes. We propose that in the presence of a TATA-box, the recognition of the TSS depends directly on a small number of TBPs and/or of TBP-related protein(s). The relaxed recognition by TBPs in TATAΔ-containing promoters might be responsible for a bias towards the large range of expression. In the absence of TATA-box or TATAΔ, the recruitment of the TBP might be mediated by different TBP-associated TFs depending on the gene. Interaction of the different TBP-associated TFs with other specific factors might therefore explain the relatively higher specificity of transcription observed for the TC_[-39,-26]_-PLM gene set.

**Table 3:** Expression of *A. thaliana *genes for the four promoter classes.

Gene sets	All genes	Only TATA-box	Only TC_[-39,-26]_-PLMs	Only TATAΔ-PLMs	_[-39,-26]_-PLM-less
HE	39.2%	**53.8% (< 1e^-30^)**	36.7% (NS)	38.1% (NS)	34.5% (1e^-22^)
LE	60.8%	46.2% (2e^-24^)	63.3% (NS)	61.9% (NS)	**65.5% (< 1e^-30^)**
SR	40.4%	41% (NS)	**44.1% (< 1e^-30^)**	37.4% (< 1e^-30^)	40.2% (NS)
WR	11.1%	10.3% (NS)	9.3% (NS)	**13.2% (< 1e^-30^)**	11.2% (NS)

WR-HE+	2.2%	2.4% (NS)	1.6% (NS)	2.2% (NS)	2.3% (NS)
WR-LE+	3.4%	2.5% (NS)	3.1% (NS)	4.0% (NS)	3.8% (NS)
SR-HE+	5.5%	**8.5% (1e^-5^)**	5.3% (NS)	5.3% (NS)	4.6% (8e^-6^)
SR-LE+	7.4%	5% (< 1e^-30^)	8.6% (NS)	6.8% (NS)	**8.1% (< 1e^-30^)**

The 8 expression categories are described in the Figure 7. HE, High Expression; LE, Low Expression; SR Small Range of expression; WR, Wide Range of expression; HE^+ ^Highest Expressions; LE^+^, Lowest Expressions. The whole gene set contains the 11161 genes for which we have both a known TSS and CATMA-expression data. We performed two one-sided Fisher exact tests allowing the identification of higher (bold) or lower (underlined) percentages in a gene set in comparison with all other genes, *i.e*. genes within the whole gene set minus genes within the considered gene set. NS indicates non-significant difference. P-values in parenthesis are less than 5% with the Bonferroni correction.

### The class of regulatory element in the [-39, -26] region is not conserved in higher plant orthologous gene pairs

With the increasing availability of transcriptome data, the search for conserved TFBSs within promoters of genes exhibiting similar transcriptional patterns has gathered much attention. Surprisingly, the conservation of core-promoter elements, widely considered as being those most conserved, has not received such large interest. One recent study addressing this question did cast doubt on this widely accepted notion [52]. Concordantly, in several studies on orthologous relationships between large gene families, we observed no significant conservation of the presence of a TATA-box between plant orthologues (data not shown). Even single copy genes present as orthologues in both *A. thaliana *and *O. sativa *are no more conserved than random pairs of genes [53]. The co-occurrence of different elements in the [-39, -26] promoter region could, at least in part, explain an apparent non-conservation at the gene level despite the observed conservation at the genomic one. This prompted us to reexamine the question comparing core-promoter elements in the [-39, -26] region in *A. thaliana *and *O. sativa*. We analysed the conservation of the three classes of _[-39,-26]_-PLMs in 5805 pairs of orthologous genes in *A. thaliana *and *O. sativa *characterized by an experimentally supported TSS. While the conservation of TATAWA appeared relatively low (17%), it was significantly higher than that expected by chance (6%) (Table 4). This was not the case for TATAΔ- or TC_[-39,-26]_-PLMs, which exhibited no conservation between orthologous genes (Table 4). Our results showing that the TATA-box is more involved at the transcriptional level and that both TATAΔ- and TC_[-39,-26]_-PLMs are more involved in specificity are in line with finding. All together our observations are in accordance with the fact that gene transcription levels are positively correlated between orthologous genes in *A. thaliana *and *O. sativa *[53] and that the correlation between transcriptional range and intensity is rather weak [51]. Nevertheless, TATAΔ- and TC_[-39,-26]_-PLMs are conserved, both at the sequence level *per se *and at the occurrence level within the whole genome.

**Table 4:** Conservation of the three [-39, -26]-PLM classes in orthologous gene pairs between A. thaliana and O. sativa.

	**Number of ortholog pairs with PLM in**			**Percentage of PLM conservation**	
	
	***A. thaliana *(a)**	***O. sativa *(b)**	**Both species (c)**	**Observed**	**Expected by chance:**
	
Only TATAWA	393	343	82	**17% (7e^-15^)**	6%
Only TATAΔ-PLMs	997	531	138	12% (NS)	11%
Only TC_[-39,-26]_-PLMs	655	602	98	13% (NS)	12%

In the 5805 pairs of orthologous genes between *A. thaliana *and *O. sativa*, we searched for the number of genes containing a given _[-39,-26]_-PLM (a) in *A. thaliana *but not in their respective *O. sativa *ortholog, (b) in *O. sativa *but not in their respective *A. thaliana *ortholog and (c) in both orthologous genes. The c/(a+c) ratio indicates the level of observed conservation for each PLM class in *O. sativa *in regard to *A. thaliana*. Then we compared the ratio to the by chance value, *i.e*. the ratio b/(5805-a-c): the presence of a given PLM in *O. sativa *orthologous genes from the PLM-less *A. thaliana *genes. We performed two one-sided Fisher exact tests allowing the identification of significantly different percentages. The underlined data indicate that the observed percentage is higher than expected. NS indicates non-significant difference. P-values in parenthesis are less than 5% with the Bonferroni correction.

## Discussion and conclusions

In *A. thaliana *and *O. sativa*, we identified a novel class of putative TFBS involving TC-elements that are preferentially located within the same core-promoter region as TATA-boxes.

We showed that these TC-elements are structurally distinct from the previously described TC-microsatellites observed in the 5'UTR of plant genes. Nevertheless, the presence of both TC-elements and TC-microsatellites in higher plants but not in vertebrates suggests an evolutionary link between these two promoter elements.

Previous reports have described the presence of a pyrimidine rich element, named the Y-patch, in plant core-promoters [21,26], and its tendency to be associated with both the TATA-box and the Inr motif [45]. Y-patch motifs and TC-microsatellites are two names used to globally describe T and C rich sequences widely surrounding the TSS. In promoters, these two elements are characterized by two non identical but overlapping groups of motifs. Our results show that the TC_[-39,-26]_-PLM exhibits specific characteristics distinguishing it from the other Y-patch motifs. Y-patch motifs show frequent occurrence in plant promoters, are present in a wide area around the TSS (see Figure 1) and may be extended from a 6- to a 10-base-long element without decreasing the score associated to their local overrepresentation (SMS). Indeed the TC_[-39,-26]_-PLMs were only observed in a sub-set of 18% of *A. thaliana *promoters at the TATA-box expected position, were associated with a sharp functional window, were 6-base-long and could not be extended without decreasing their SMS.

Specific promoter features were frequently observed when genes were classified into groups, *i.e*. the TC-element-containing gene group, the TATA-box-containing gene group or the TATAΔ-PLMs-containing gene group. First, our data indicate that the TATA-box preferentially associates with a CA-TSS. Neither the TATAΔ- nor the TC-elements exhibited this apparent functional association. In addition, no association between the TATA-box and any of the three other YR-TSS was observed. Second, TC-element-containing genes were predominately large and involved in protein metabolism while TATA-box-containing genes were predominantly compact and involved in response to stress and stimulus. Indeed, gene function, specificity and level of expression are linked features. Using an original approach we have been able to distinguish the possible involvement of different _[-39,-26]_-elements in the control of either the level or the specificity of expression. A global analysis of CATMA-transcriptome data indicated preferential links between the presence of: (i) a TATA-box and high gene expression; (ii) TC-elements and high specificity of gene expression; and (iii) TATAΔ-PLMs and broad expression patterns, as in housekeeping genes. All together our observations suggest that TC-elements might be considered as a novel class of plant promoter elements linked directly or indirectly to the regulation of gene expression.

The presence of all three elements, *i.e*. TATA-boxes, TC- and TATAΔ-elements, have been observed in two plants that diverged about 150 millions of year ago [29]. We observed a global conservation, *i.e*. conservation in the relative number of genes containing one of the three motifs in the two genomes. Nevertheless, only the TATA-box showed a low level of conservation between orthologous gene pairs while conservation of either TC- or TATAΔ-elements between orthologous gene pairs was no higher than that found between any gene pair. On the one hand, the low level of conservation at the orthologous gene level is in line with the rapid evolution of core regulatory motifs after gene duplication in *A. thaliana *[43] and with the notion that *A. thaliana *and *O. sativa *may have independently evolved novel TSSs [54]. On the other hand, the conservation of the relative number of genes with either a TATA-box, a TC- or a TATAΔ-element suggests a global conservation of the interspecies variability, noise level and evolvability of gene expression; three processes involving TATA sequences [55-57]. The three classes of core-promoter elements observed in the [-39, -26] region are each present in about 20% of the promoters. In this study, we used "clean" classes of promoters, *i.e*. those containing only one of the three classes of elements. However in several other promoters, more than one element from a given class may be present. Therefore many functional combinations are expected. Experimental data have shown that the TATA-box is involved in the direct binding of TBP [5] and at least some TC- and TATAΔ-elements might be involved in the recognition by other TFs recruiting TBP to form the transcription initiation complex at the right position. Consistent with this notion, both TC- and TATAΔ-elements are linked to the specificity of expression that could be mediated through different TFs under the control of various signals. An alternative would be that TATA-boxes, TATAΔ-elements and TC-elements are all responsible for DNA instability around -30 relative to the TSS but at different levels [25] promoting a continuous range of control on gene transcription. Either way, we believe that our observations call for further biological analysis and that our predictions will be useful for future assessment of the relationships between promoter architecture and gene expression. In this respect, future studies in model plants should be based on a better description of the different organization of core-promoters and include a more precise definition of the location of alternative TSSs based on new sequencing technologies.

## Methods

### Promoter sets

*Arabidopsis thaliana *and *Oryza sativa *promoters were built from transcripts, Expressed Sequence Tags and full-length cDNA (The *Arabidopsis *Information Resource - TAIR R.6 [41] and The Institute for Genomic Research - TIGR R.3 [58] respectively). We used FLAGdb^++ ^[59], an integrative database of plant model genomes, to define the transcriptional units by aligning all the available transcripts to gene models, excluding pseudogenes. We excluded promoters with a 5'UTR smaller than 50 bases in order to avoid truncated UTRs. Our promoter sets included 14927 *A. thaliana *and 18012 *O. sativa *sequences extending 1 kb upstream of the predicted TSS and containing the whole 5'UTR. The 15802 *Homo sapiens *and the 15833 *Mus musculus *promoters were retrieved from the DBTSS [39]. To maintain a consistent approach to that used to construct the plant promoter set, we selected only the TSSs located the most upstream of genes.

### Identification of Preferentially Located Motifs

For each motif analysed, we extracted all occurrences within a promoter set. The motif position corresponds to the position of the first base of the sequence. Motif distributions were determined using a one-base-long sliding-window with a one-base shift. For each window, we counted the number of promoters containing the motif rather than the number of occurrences to avoid favouring repeated motifs. The promoter sequences were then divided into two regions. First, the [-1000, -300] region was used to learn the distribution model using a simple linear regression and determine a 99% confidence interval. Second, within the [-300, 500] region, we searched for non-evenly distributed motifs, *i.e*. those exhibiting a peak above the confidence interval. We increased the sliding-window size from 1 to 100 bases step-by-step when a second peak was detected or when the learning area contained at least one window within which no motif was detected, in order to avoid non-accurate learning of the distribution model.

For each PLM we recorded: (i) the motif distribution, (ii) the window size, (iii) the preferential position, *i.e*. the position of the peak top in the distribution, (iv) the peak boundaries allowing the functional window to be located, (v) the list of promoters containing the motif and (vi) the Score of Maximal Square relative to the base line or SMS, *i.e*. the ratio (peak top minus base line)/(upper bound of the confidence interval minus base line). We only considered PLMs characterized by an SMS greater than 1.

### Sequence distance graph and seed motifs

Out of all _[-39,-26]_-PLMs, we searched for those differing by a single substitution and represented the links by means of a graph. For both TA-PLM and TC-PLM graphs we identified the seed(s), *i.e*. PLM(s) only connected in the graph to PLMs less present in promoters than itself. The graph is thus oriented relative to the seed(s). The TA-PLM graph comprises the TATA-box and all the 15 TATAΔ-PLMs. The TC_[-39,-26]_-PLM graph was first constructed with the PLMs observed in more than 200 promoters. Then, remaining PLMs were added considering only links with the PLMs making up the graph and not those with the remaining PLMs.

### Extended motif analyses

PLM relevance is ranked by the SMS value. For each PLM, we analyzed the SMS of all motifs extended by one base upstream or downstream of the initial PLM sequence. We referred to these motifs as extended-motifs. Selected extended-motifs were only those that (i) were a PLM, (ii) shared the initial PLM constraints, *i.e*. a sharp peak in the [-39, -26] region and (iii) were characterized by an SMS 1.1-fold higher than that of the initial PLM, *i.e*. exhibited a stronger topological constraint.

### Gene structure and gene function

All information about gene structure was obtained from FLAGdb^++ ^[59] including: (i) the median of the lengths of frames, coding sequences (CDSs), 3'UTRs and 5'UTRs, first introns and cumulated introns; and (ii) the percentage of intron-less genes.

Information about gene function was obtained from the TAIR Gene Ontology (GO) categories [41,60]. We used the GO Biological Function and GO Cellular Component categories. For a given gene set, we calculated the percentage of genes within that category relative to the remaining *A. thaliana *genes with an experimentally supported TSS. Finally, we only considered previously annotated genes, *i.e*. we excluded the "unknown" and the "other" categories.

### Gene expression

The transcriptome data used in this work were obtained using the CATMA v2 microarray [61]. They include 522 hybridized samples extracted from 40 different projects covering 12 organ types. Expression data were downloaded from CATdb [50] containing all the transcriptome data generated by the CATMA-URGV platform. They had also been deposited in either the NCBI Gene Expression Omnibus [62] or the European Bioinformatics Institute ArrayExpress [63] repositories. Out of the 14927 *A. thaliana *genes with an experimentally supported TSS, we analyzed the expression data of 11161 genes specifically relating to one CATMA probe. Each gene was spotted according to (i) the percentage of samples in which it had been detected, called the hybridization percentage and (ii) its median expression level. We performed a third order linear regression allowing us to cluster 4371 genes with a High level of Expression (HE) and 6790 with a Low level of Expression level (LE). Different hybridization ranges were also defined according to two inflections in the linear regression curve. The Small Range (SR) class included 4510 genes characterized by a hybridisation percentage of less than 15% and the Wide Range (WR) class included 1241 genes characterized by a hybridisation percentage of over 85%. Second, we built a 60% confidence interval allowing the identification, within each class, of the highest and the lowest expression levels (respectively HE+ and LE+).

### Orthologous genes

We selected those *A. thaliana *and *O. sativa *orthologous gene pairs being Bidirectional Best Hits [64] with BLASTp [65] resulting in 5805 orthologous pairs of genes having an experimentally supported TSS. The presence of a PLM in both genomes led to the distinction between the presence of this PLM within (i) *A. thaliana *promoters but not in their respective *O. sativa *orthologues; (b) *O. sativa *promoters but not in their respective *A. thaliana *orthologues; or (c) both orthologous genes. For each PLM-class, we performed comparisons between expected and observed conservation within orthologous gene pairs. The c/(a+c) ratio indicates the level of observed conservation in *O. sativa *with respect to *A. thaliana*. The expected conservation value is given by the ratio b/(5805-a-c), *i.e*. the presence of a given PLM-class in *O. sativa *orthologous genes from the PLM-class-less *A. thaliana *genes.

### Statistical analysis

Statistical analyses were performed with the R statistical software [66]. We used R for (i) the regression analysis leading to PLM identification (Figure 1) and the characterization of expression categories (Figure 7), and for (ii) motif distributions (Figures 3 and 6). We performed Fisher exact one-sided tests using the Bonferroni correction to compare percentages between two independent samples. We searched for statistical decreases or increases in the percentages of one set of genes (i) in GO annotation categories (Table 1), (ii) in intron-less (Table 2, last row), (iii) in expression categories (Table 3) and (iv) with a given motif (Table 4). Structural gene features, even after log modification, cannot be assumed to be normally distributed. We therefore performed Wilcoxon-Mann-Whitney one-sided tests using the Bonferroni correction to compare two independent structural gene feature distributions. We considered the hypothesis that structural data might result in higher or lower values for a given gene set compared to all other genes, *i.e*. the 14927 genes minus the considered gene set (Table 2, first rows). Each p value less than 5% with the Bonferroni correction was considered significant.

## Abbreviations

CATMA: Complete *Arabidopsis *Transcriptome MicroArray; TFBS: Transcription Factor Binding Site; GO: Gene Ontology; HE: High Expression; LE: Low Expression; PLM: Preferentially Located Motif; SMS: Score of Maximal Square relative to the base line; SR: Small Range; TBP: TATA-binding protein; TSS: Transcriptional Start Site; WR: Wide Range;

## Authors' contributions

VBe performed the analyses and wrote the manuscript. VBr coordinated the analyses and helped to write the manuscript. AL managed the study and helped to write the manuscript. All authors read and approved the final manuscript.

## Supplementary Material

Additional file 1 **Effect on the score of an extension by pyrimidines of TC_[-39,-26]_-PLMs**. First column, motifs that do not exhibit an increase in their SMS (in parentheses) when extended. Second column, motifs that, when extended exhibit an increase in their original SMS due to the generation of an other 6-base-long motif (underlined sequence) characterized by a higher SMS (underlined SMS). Note that the SMS of the 7- or 8-base-long motifs are all lower than the SMS of the underlined 6-base-long motifs. Third column, motifs that can be extended in 7- or 9-base-long motifs. Note that the two long extensions are made of three repeats of TCT. Click here for file
